# Environmental cycles regulate development time via circadian clock mediated gating of adult emergence

**DOI:** 10.1186/s12861-018-0180-6

**Published:** 2018-12-22

**Authors:** Manishi Srivastava, Anjana James, Vishwanath Varma, Vijay Kumar Sharma, Vasu Sheeba

**Affiliations:** 1 Chronobiology Laboratory, Evolutionary and Integrative Biology Unit (Formerly Evolutionary and Organismal Biology Unit), Jawaharlal Nehru Centre for Advanced Scientific Research, Bangalore, Karnataka India; 2 Behavioural Neurogenetics Laboratory, Neuroscience Unit, Jawaharlal Nehru Centre for Advanced Scientific Research, Bangalore, Karnataka India

**Keywords:** Pre-adult development, Emergence, Pupariation, Circadian clocks, *Drosophila*, *Period*, Adult emergence rhythm, Pupation rhythm

## Abstract

**Background:**

Previous studies have implicated a role for circadian clocks in regulating pre-adult development of organisms. Among them two approaches are most notable: 1) use of insects whose clocks have different free-running periods and 2) imposition of artificial selection on either rate of development, timing of emergence or circadian period in laboratory populations. Using these two approaches, influence of clock on rate of development has been elucidated. However, the contribution of circadian clocks in determining time taken for pre-adult development has remained unclear. Here we present results of our studies aimed to understand this influence by examining populations of fruit flies carrying three different alleles of the *period* gene and hence having different free-running periods. We tried to achieve similarity of genetic background among the three strains while also ensuring that they harbored sufficient variation on loci other than *period* gene.

**Results:**

We find that under constant conditions, flies with long period have slower development whereas in presence of light-dark cycles (LD) of various lengths, the speed of development for each genotype is influenced by whether their eclosion rhythms can entrain to them. Under LD 12:12 (T24), where all three strains entrain, they do not show any difference in time taken for emergence, whereas under LD 10:10 (T20) where long period flies do not entrain and LD 14:14 (T28) where short period flies do not entrain, they have slower and faster pre-adult development, respectively, compared to the controls. We also show that a prior stage in development namely pupation is not rhythmic though time taken for pupation is determined by both the environmental cycle and *period* allele.

**Conclusion:**

We discuss how in presence of daily time cues, interaction of the cyclic environmental factors with the clock determines the position and width of the gate available for a fly to emerge (duration of time within a cycle when adult emergence can occur) resulting in an altered developmental duration from that observed under constant conditions. We also discuss the relevance of genetic background influencing this regulation.

**Electronic supplementary material:**

The online version of this article (10.1186/s12861-018-0180-6) contains supplementary material, which is available to authorized users.

## Background

Circadian clocks, innate 24 h time keeping mechanisms, coordinate behavioral and physiological processes of organisms by keeping them in-sync with environmental time cues. In fruit flies *Drosophila melanogaster,* the most commonly studied circadian behaviors are locomotor activity rhythms and adult emergence rhythms. While locomotor activity rhythms can be studied for individual flies, adult emergence, which is a one-time occurrence in the life of an individual fly, shows rhythmicity at the population level and is known to be gated by circadian clocks [1, 2].

In *Drosophila,* adult emergence shows persistent rhythmicity [3] with a large proportion of emergence occurring during daytime and the emergence peak occurring shortly after dawn [4]. Early studies on the circadian control of eclosion in *Drosophila melanogaster* showed that even though the intermediate steps of development are not under circadian control, the phenomenon of adult emergence is rhythmic [5–7]. The circadian control of eclosion was further elaborated through studies which showed an interaction between prothoracic gland which regulates developmental state and the circadian clock [8]. Prothoracic gland secretes ecdysone, a steroid hormone which regulates molting and metamorphosis and activity of this gland can also influence the periodicity of emergence rhythm [9]. Oscillations of clock proteins observed in prothoracic gland under light-dark cycles have been found to require inputs from clock neurons in the brain in order to persist under constant conditions [8]. Such input from the circadian clock to the prothoracic gland maybe via Prothoracicotrophic Hormone (PTTH) which also sets the duration of feeding interval as demonstrated by delayed larval development and increased size of adults that emerge in absence of PTTH [10]. Additionally, PTTH plays an important role in coordinating environmental cues and the developmental process and therefore has an influence on adaptive plasticity of the developmental program [11]. Furthermore, core clock genes such as *period* and *timeless* are required for rhythmicity in eclosion [1, 9] and are also essential for development as they are required for transcriptional upregulation of enzymes needed for production of steroids that play an important role in larval development [12]. Thus, there are multiple, intricate connections between circadian clock, steroidal hormones and developmental processes which interact to regulate timing of adult emergence.

Apart from governing the rhythm in emergence of an adult fly from its pupa by gating it as discussed above, clocks are also considered to have an effect on the overall rate of pre-adult development. Circadian clocks are known to be functioning from early developmental stages in other organisms like zebrafish and mice [10, 11] and also affect differentiation and proliferation of adult stem cells [12] though there is not much clarity in their role in embryonic development [13]. In *Drosophila,* clocks are known to be functional even during hatching [14] and a relationship between clock and pre-adult development was first established by a study on the *period* gene mutants where a positive correlation was seen between clock period and pre-adult development time [15]. It was demonstrated that while short period (*per*^s^) mutants tended to show shorter overall pre-adult development, long period mutants (*per*^*l*^) showed a longer development time in terms of time taken for pupation as well as emergence (with slight differences between the pupation and emergence profiles). However, since the correlation between period and development time persisted even under constant light (LL, where many behavioural patterns of flies are rendered arrhythmic and core clock oscillations are expected to be disrupted), it is not possible to conclude that the differences in developmental rate are mediated by the circadian clock per se. Hence, the contribution of circadian clocks in determining development time differences remains ambiguous. Additionally, since mutant fly lines are typically highly inbred and could generate spurious genetic correlations between fitness components, they are not ideal for examining relationships between critical life-history traits like development time and the circadian clock [16]. Another study using wild type flies (*per*^*+*^) and *per*^*s*^ mutants established that the clock assesses developmental state a few hours prior to eclosion, which results in determination of the gate chosen for eclosion as well as the timing of emergence within that gate [17]. Moreover, in *Drosophila melanogaster* populations subjected to T-cycles of increasing period lengths, a positive correlation was seen between the rate of pre-adult development and the length of the external light-dark cycle [18]. Besides suggesting a possible role for the clock in gating of eclosion and in the regulation of pre-adult development, this study also concluded that differences in development time between clock mutants may not be due to pleiotropic effects since flies with similar free-running period (~ 24 h) exhibited different rates of pre-adult development under external cycles of different periodicities. However, it can also be speculated from these results that the period of external cycle is a more important determinant of the rate of pre-adult development than the internal clock period. Moreover, these results were not in concordance with the previous study using *period* gene mutants [15] where it was shown that irrespective of external conditions, the positive correlation between the free-running period of the clock and the mean development time is maintained therefore implying that the external conditions have no influence on the regulation of development by the circadian clock.

The above studies lead to contradictory conclusions but are not directly comparable due to the difference in their approaches, the former used mutant fly lines with divergent intrinsic period while the latter used wild type flies and subjected them to extreme T-cycles. Therefore to examine the relative roles of circadian clock and external cyclic environment on the speed of pre-adult development and gating of emergence in *D. melanogaster* we conducted a systematic study by first generating a set of three populations of fruit flies (Additional file 1), two of them carrying either the short or long mutant alleles of the *period* gene (*per*^*s*^ and *per*^*l*^ respectively) while the third is expected to carry a wild type copy of the same (*per*^+^). The mean free running period of these *per*^*s*^*, per*^*+*^ and *per*^*l*^ lines are 18.58, 24.17 and 28.70 h respectively (Additional file 2). To verify whether pre-adult development time (measured as total time taken from egg collection to emergence) may be altered by merely harboring a certain allele of the *period* gene, we first assayed the three fly lines in constant darkness (DD) and constant light (LL) where external cycles cannot play a role. Further, in order to understand the interaction between internal and external rhythms’ period and their respective roles in determining development time, we assayed time to emergence across different T-cycles (length of external light-dark cycle) as well. Using this approach, we had combinations of different internal and external periods. We have seen in an independent study that *per*^*s*^ and *per*^*l*^ flies show differences in the range of entrainment of their activity rest rhythm (Manishi Srivastava, Vishwanath Varma, Abhilash Lakshman, Vijay Kumar Sharma and Sheeba Vasu, *unpublished manuscript*) where *per*^*l*^ flies do not entrain to a light-dark cycle of 20 h duration, while both *per*^*s*^ and *per*^*+*^ entrain to a wide range of T-cycles. Therefore, we first assessed the entrainability of adult emergence rhythms of these three strains under three different T-cycles. We speculated that if the internal clock has a major role to play in regulating time to emergence, the trend across strains would further vary depending on whether a strain can entrain to that regime i.e. the strains which entrain will show similar time to emergence whereas the one that does not might have a different time to emergence.

We show an association between intrinsic period and time to emergence, and further demonstrate that even under the influence of external cycles the variation in time to emergence is dependent on the intrinsic period of the clock as flies which do not entrain are not bound by the eclosion gate imposed by the light-dark cycle and therefore overall development time is different from those that are entrained.

## Results

### Flies with different free-running periods exhibit different development time under constant conditions

**Fig. 1:**
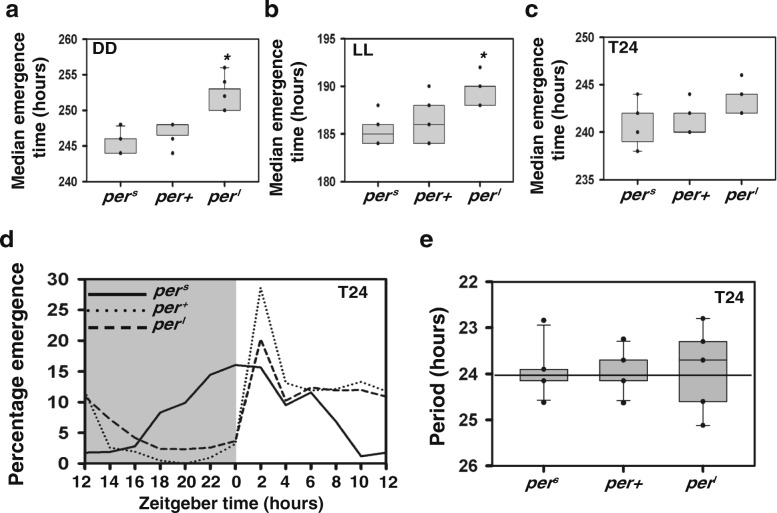
Emergence time under constant conditions and LD 12:12. (**a**-**c**) Box plots show the median time to emergence for each vial and strain (*n* > 8 vials; 30 eggs/vial) under three regimes. Whiskers extend up to highest and lowest values and dots show the individual data. Under (**a**) constant darkness (DD) at 25 °C and (**b**) constant light (LL) at 25 °C asterisks show that *per*^*l*^ differs significantly from other strains (*p* < 0.05) based on Kruskal-Wallis test for multiple independent samples. (**c**) LD 12:12 (T24) at 25 °C. (**d**) Adult-emergence profiles of the three strains (*n* = 10 vials; 300 eggs/vial) under T24 where percentage emergence is plotted against zeitgeber time, 0 being the time of lights-on. Shaded region represents duration of the LD cycle during which lights were off. (**e**) Box plots show period values obtained by COSINOR analysis for each strain under T24. Whiskers extend up to highest and lowest values and dots show the individual vial median data points. Black line depicts the period of T-cycle

We first compared the average pre-adult developmental time (measured as time from egg collection to emergence) across the three strains under 25 °C in absence of external cycles. Under DD, median emergence time across strains was ~ 250 h. Kruskal-Wallis test for medians showed that time to emergence for *per*^*l*^ flies was significantly longer when compared to *per*^*s*^ and *per*^*+*^ (*p* < 0.05; Fig. 1a) while there was no statistically significant difference between *per*^*s*^ and *per*^*+*^. These results differ from that in a previous study [15] where all three fly strains showed significantly different time to emergence as well as pupation from each other under constant as well as entraining conditions.

Under LL, as previously reported [17, 18], the median time to emergence was much shorter (~ 185 h) than what was observed under DD for all the three strains with a similar trend across the strains as in DD. The trend observed was similar to that under DD i.e., significantly larger time to emergence for *per*^*l*^ flies when compared to *per*^*s*^ and *per*^*+*^ (*p* < 0.05; Fig. 1b) while there was no statistically significant difference seen between *per*^*s*^ and *per*^*+*^. Thus, we find that in the absence of daily time cues, irrespective of DD or LL conditions, time to emergence of *per*^*l*^ flies is longer than the other two strains suggesting that the *per*^*l*^ allele causes a delay in the completion of the developmental process.

### Time taken for adult emergence varies depending on entrainability to light dark cycle

Next, we asked how environmental cycles might alter developmental rate and whether interactions between entraining conditions and internal clock affect developmental rate. We assessed time to emergence under symmetric light-dark cycles of different cycle lengths (T-cycles) along with adult emergence rhythms of the three strains under the same conditions. Pairwise comparison using Kruskal-Wallis test for medians showed no significant difference in time to emergence among the three strains under T24 (*p* > 0.05; Fig. 1c) when assayed at a density of 30 eggs/vial. Under this regime, we also separately analyzed time to emergence of males and females and found emergence of roughly equal numbers of males and females across vials and that both males and females of the three strains also do not show significant differences in time to emergence (*p* > 0.05; Additional file 3 a, b, Table 1) like the combined data shown in Fig. 1c. We also compared time to emergence of arrhythmic *per*^*0*^ mutants with wild type *per*^*+*^ in this regime and found that *per*^*0*^ flies had significantly shorter time to emergence compared to *per*^*+*^ (*p* < 0.05; Additional file 4) i.e., they emerged sooner than controls suggesting that in wild type flies, *period* gene mediated gating of developmental processes delays adult emergence.

**Table 1:** Table shows vial-wise ratios of males/females emerged obtained for the three strains under LD 12:12

	*per* ^*s*^	*per* ^+^	*per* ^*l*^
V1	0.92	0.79	1
V2	0.92		0.9
V3	0.5		0.8
V4	1.09	0.9	1.43
V5	0.53	0.67	1.08
V6	1	0.73	1.89
V7	1.2	1.67	0.67
V8	0.67	1.08	1.18
V9	1.08	1	
V10	0.1	0.41	0.86

**Fig. 2:**
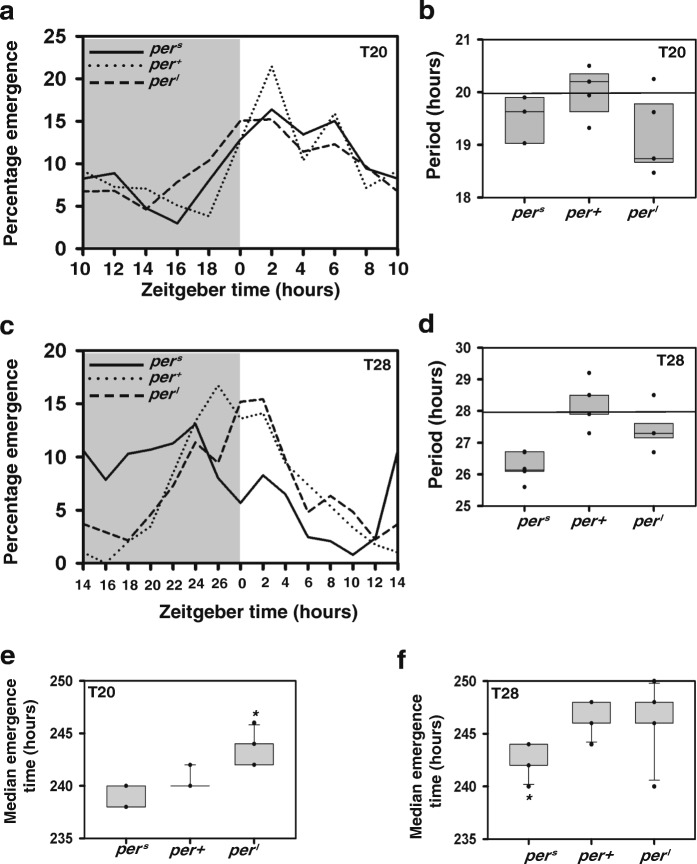
Emergence time under deviant T-cycles. (**a**) Average adult-emergence profiles of the three strains (n > 8 vials; 300 eggs/vial) under T20 averaged across cycles where percentage emergence is plotted against zeitgeber time. (**b**) Box plot shows period values obtained by COSINOR analysis for each strain under T20. (**c**) Adult-emergence profiles of the three strains (n > 8 vials; 300 eggs/vial) under T28 averaged across cycles where percentage emergence is plotted against zeitgeber time. (**d**) Box plot shows period values obtained by COSINOR analysis for each vial under T28. (**e**) Median pre-adult emergence time for each strain (*n* > 9 vials; 30 eggs/vial) calculated as average of medians across vials when assayed under LD 10:10 (T20) at 25 °C. Asterisk shows that *per*^*l*^ differs significantly from other strains (*p* < 0.05) based on Kruskal-Wallis test for multiple independent samples. (**f**) Median pre-adult emergence time for each strain (n > 9 vials; 30 eggs/vial) calculated as average of medians across vials when assayed under LD 14:14 (T28) at 25 °C. Asterisk shows that *per*^*s*^ differs significantly from other strains (*p* < 0.05) based on Kruskal-Wallis test for multiple independent samples. All other details are similar to Fig. 1

Adult emergence rhythms of all the three strains were assayed by collecting eggs at a density of 300 eggs/vial (in order to obtain multiple cycles of emergence), to determine whether a given regime was capable of entraining the circadian clock. We found that they entrained under T24 (Fig. 1d) as their periodicities were not significantly different from 24 h (*p* > 0.05; Additional file 5, Fig. 1e). Under T20, *per*^*l*^ did not entrain (Fig. 2a) i.e., they exhibited periodicity significantly different from 20 h (*p* < 0.05; Additional file 6, Fig. 2b) whereas the other two strains entrained with periodicity not significantly different from 20 h (*p* > 0.05). Similarly, under T28, *per*^*s*^ did not entrain (Fig. 2c), showing periodicity significantly different from 28 h (*p* < 0.05) which was not the case for other two strains (*p* > 0.05; Additional file 7, Fig. 2d).

In accordance with the results obtained for emergence rhythms, under T20, time to emergence of *per*^*l*^ flies was significantly longer when compared to *per*^*s*^ and *per*^*+*^ (*p* < 0.05) while *per*^*s*^ and *per*^*+*^ flies did not differ from each other (*p* > 0.05; Fig. 2e). Under T28, *per*^*s*^ flies took significantly lesser time to emergence compared to *per*^*+*^ and *per*^*l*^ (*p* < 0.05) while *per*^*l*^ and *per*^*+*^ did not differ significantly from each other (*p* > 0.05; Fig. 2f). Across T-cycles comparisons for total time to emergence using Kruskal-Wallis test showed a significant main effect of regime on median emergence time for each strain (*p* < 0.05), when assayed for time to emergence, with a trend of increase in time to emergence with increase in length of T-cycle. Thus, we show that rate of pre-adult development is dependent on the length of the external light-dark cycle and flies which are able to entrain to a given T-cycle have comparable time to emergence.

### Strain differences exhibit divergent trends in pupation and emergence time

**Fig. 3:**
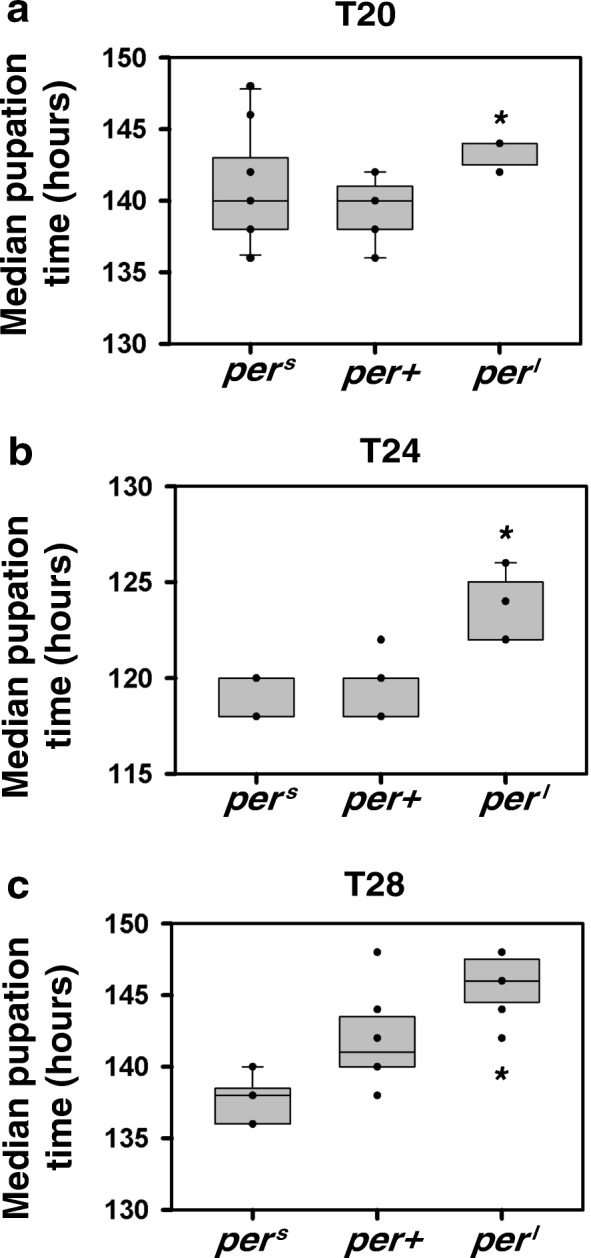
Pupation time under deviant T-cycles. Box plots depict the median pupation time for each strain (n > 9 vials; 30 eggs/vial) calculated as average of medians across vials when assayed under (**a**) LD 10:10 (T20) (**b**) LD12:12 (T24) and (**c**) LD 14:14 (T28) at 25 °C. Asterisk shows that *per*^*l*^ differs significantly from other strains (*p* < 0.05) based on Kruskal-Wallis test for multiple independent samples. All other details are similar to Fig. 1

Since it is possible that environmental cycles and/or the circadian clock may act on some prior developmental stages such as pupariation, and the final act of emergence may be simply a reflection of gating at earlier stages, we compared time to pupation for the three strains under the three T-cycles examined and compared the trends among strains with time to emergence. Kruskal-Wallis test for medians showed that pupation time under T20 and T24 for *per*^*l*^ flies was significantly longer when compared to *per*^*s*^ and *per*^*+*^ (*p* < 0.05; Fig. 3a, b) while there was no significant difference between *per*^*s*^ and *per*^*+*^ (*p* < 0.05; Fig. 3a, b). Under T28, all three strains were significantly different from each other (*p* < 0.05; Fig. 3c). We find that although the trends among strains for pupation and emergence are similar under T20 (compare Fig. 2e and Fig. 3a), the trends were not consistent among strains across the two stages of pupation and development under T28 (compare Fig. 2f and Fig. 3c) and T24 (compare Fig. 1c and Fig. 3b). Since we hypothesized that the presence or absence of differences between strains in emergence time depended on gating by the external cycle under entrained conditions, we asked whether pupation also is gated by the external cycle. In a separate study assaying the pupation rhythm of a related ancestral population, we found that wild type flies do not pupate rhythmically under T24. One-way ANOVA on pupation profile across cycles revealed no significant effect of time point (*p* > 0.05; Additional file 8). Thus, we find that pupation is not rhythmic and does not correspond with time to emergence in all regimes.

## Discussion

Rate of development is an important life-history trait that may be a critical contributor to reproductive fitness in insects [16]. Since the circadian clock gates adult emergence and influences developmental rate, an understanding of the role of clocks in this regulation of pre-adult development is important. Although a few studies have shown an effect of clock period on development time, there is no clarity on the extent to which this regulation is imposed by the internal clock/external cycle and to what characteristics of the clock this role can be attributed.

We saw that, under DD, *per*^*l*^ flies had significantly longer time to emergence when compared to *per*^*s*^ and *per*^*+*^ flies (Fig. 1a). However, *per* ^*s*^and *per*^*+*^ flies did not differ from each other. This is in contradiction to what was seen in the previous study with *period* mutants, where all the three strains differed significantly from each other [15]. This difference in the observation across the two studies can be attributed to differences in genetic backgrounds of the flies used for the two studies. While the previous study was done on mutant and wild type lines that were backcrossed with a line carrying a deficiency on X chromosome, which was likely to be highly inbred, in our study all the *period* alleles have been backcrossed to a highly outbred, wild-type population which is therefore less likely to have undergone genetic drift and hence fixation of alleles at large number of loci in the genome. Since any phenotype affected by a mutation is also likely to have effects of the interactions of that mutation with other genes [19], there is a possibility that the difference which was observed previously between *per*^*s*^ and *per*^*+*^ flies was a consequence of interactions of the mutated clock genes with the specific genetic background.

We find that under LL, although time to emergence was relatively shorter compared to DD, the differences across three strains were similar to that under DD i.e. *per*^*l*^ flies had a slower developmental rate (Fig. 1b). Therefore, our observation of faster development under LL compared to DD is in concordance with the previous studies on mutants as well as on the other wild type populations [18, 20]. We concur with previous reports [18] that under LL, an absence of gating for eclosion results in this acceleration. Thus, we can say that the inherent differences observed across the three strains are maintained when there are no time cues present and even when the clock may have been rendered dysfunctional and therefore there is a dependence of time to emergence on the free-running period, genetic background and the limitations imposed by the gates available for adult emergence. Correlations between period and rate of development have also been observed in studies where large, outbred, replicate laboratory populations with sufficient genetic variation were used for imposing artificial selection. Selection on phase of emergence as well as on pre-adult development time has been shown to result in correlated responses in the clock’s period. Selection on early and late phase of adult emergence yielded concomitant decrease and increase in pre-adult development time of *Drosophila* populations [20]. Clock period and development time were also found to be correlated in some other laboratory selection studies where fly populations were selected for faster or slower development. An increase in rate of pre-adult development as a result of artificial selection also resulted in the shortening of the free-running period in most cases [21–23]. Thus, correlations between period and pre-adult development have been observed through mutant lines as well as artificially selected populations.

The pupation results from our fly lines show a general delay in rate of pupation with increase in period in each of the three T-cycles (Fig. 3) which does not correspond to the trends observed in time to emergence. However, this is not surprising as assay of percentage pupation across multiple cycles did not reveal a significant main effect of time point which indicates that gating by external cycle does not influence the rate of pupation but occurs only later during emergence, thereby altering the total time taken to emergence. Hence, it appears that the internal clock does have an effect on developmental rate, though the timing of emergence is determined by the circadian gate imposed by the external cycle when the fly is entrained to it.

To verify that these differences are clock mediated, we modified the period of the clocks by subjecting them to light-dark cycles of different durations. Since all clock-regulated processes are believed to be accelerated with an increase in the speed of the clock, development time should also conform to such expectations if it is under circadian control. Hence, we used an approach of altering the period of the clock as well as the zeitgeber to study the relationship between developmental rate and clock. Time to emergence increased with an increase in the length of zeitgeber. This observation is in concordance with a previous report of flies with ~ 24 h period showing an increase in duration of development time with increase in length of light-dark cycle [18] and thus demonstrates that influence on rate of development by clock genes is not mediated by pleiotropic effects.

In the adult emergence assay conducted across T-cycles of different durations, we see that *per*^*s*^ flies entrain under T20 and T24 regimes but not under T28 whereas *per*^*l*^ flies entrain under T24 and T28 conditions but not under T20 (Fig. 2a-d). On the other hand, wild type flies (*per*^*+*^), which are employed as controls in our case, show entrainment under all the three T-cycles (Fig. 1e, Fig. 2b, d) as also previously discussed [24]. We see that difference in time to emergence which is observed across flies with different free-running periods under constant conditions is absent under LD12:12 (Fig. 1c). Also, *per*^*0*^ flies differed significantly when compared with *per*^*+*^ in time to adult emergence and emerged sooner than controls (Additional file 4). The adult emergence rhythms of these loss-of-function mutants have been shown to be arrhythmic even under laboratory LD 12:12 conditions [25]. Therefore, it is possible that due to an absence of gating by the internal clock, *per*^*0*^ flies were able to emerge earlier compared to wild type flies which entrained to LD 12:12 and therefore were bound to emerge only within the gates imposed by their entrained clocks.

Our finding under T24 also differs from what was previously reported in *Drosophila* populations subjected to an artificial selection for timing of emergence where close association between phase of emergence and mean development time in the early and late chronotypes (which have different free running periods) was observed [20]. However, the evolved differences in development time of early and late populations in that study could not be attributed to circadian gating since this difference persisted not only under entrained but also LL conditions. In the present study, we have used an approach to minimize the influence of any factor other than gating imposed by the internal and external cycle on the rate of development. Therefore, we attribute the absence of a difference in the pre-adult development time among the three strains in LD 12:12 to the fact that all flies could entrain to this particular regime and thus they were bound to emerge in the same gate. Under short T-cycle (T20), we show that the developmental rate of *per*^*l*^ flies is slower compared to *per*^*s*^ and *per*^*+*^ whereas under long T-cycle (T28) that of *per*^*s*^ is faster compared to *per*^*+*^ and *per*^*l*^ (Fig. 2e, f). This is in concordance with our expectations that flies which entrain to a particular regime would be bound to emerge in the same gate whereas those that do not entrain could emerge according to gates of their free-running eclosion rhythm or could be subject to phenomena such as relative co-ordination. Thus, these sets of experiments confirm that the gating of eclosion, which depends on the ability of a fly to entrain to a given T-cycle is the major determinant of total time to emergence. It shows that development time is dictated by the environment only if the environmental cycle is able to entrain the circadian clock. Further, experiments which examine other loss of function mutants of the circadian clock using a similar approach would add credence to the above conclusion.

## Conclusion

Our results provide further evidence for clocks playing a significant role in the rate of pre-adult development under constant as well as entraining conditions. We demonstrate that free-running period is associated with overall time to emergence. Additionally, timing of emergence can be further influenced by external cycle by altering the timing or duration of the gates that are available for the adult fly to emerge from its pupa. Also, we emphasize the contributions of the genetic background on the observed relationships between internal clock period and time to emergence.

## Methods

### Fly lines and maintenance

Mutant lines *per*^*s*^ (~ 18 h) and *per*^*l*^ (~ 28 h) (originally obtained from Jeffrey Hall’s lab, Brandeis university, USA maintained in our laboratory for approximately 6 years) were backcrossed for five to seven generations to a wild type outbred population, *per*^*+*^ (~ 24 h). Using a large outbred population of *Drosophila melanogaster* [26], individuals carrying either the short or long period mutation, *per*^*s*^ or *per*^*l*^*,* were backcrossed for seven generations to create two lines each carrying a short or long period allele of the *period* gene in an otherwise similar genetic background under the assumption that five generations of back crossing should result in similarity of the background [27]. The backcrossing scheme is depicted in supplementary material (Additional file 1a, b). Another arrhythmic strain i.e. *per*^*0*^ was backcrossed for five generations to *per*^*+*^ and compared with the latter under T24*. per*^*+*^ was previously a population obtained by mixing four replicates of an outbred population used as controls [25]. One of these control populations (CP1) [28] were also employed in this study to examine rhythmicity in pupation. The backcrossed lines have been maintained in cages on a 21 day generation cycle under LD 12:12 at constant temperature of 25 °C and controlled humidity with banana-jaggery food provided ad libitum on alternate days. The frequency distribution of free-running period under DD at 25 °C in the three strains is shown in Additional file 2. Cages were provided with yeast paste for two days prior to egg collection for the experimental setups. Two kinds of experiments, 1) to assay time to emergence/pupation (two different experiments) and 2) to examine rhythm in emergence were conducted separately with different egg densities as described below.

### Development-time assay

Prior to egg collection a dummy food plate was placed in the cage populations for 1 h which allowed flies to lay any eggs that may have been retained within the female (Sellier, 1955, referred in [29]). This dummy plate was replaced by another food plate on which flies laid eggs for two hours and these eggs were collected for the assays (similar to [30]). Exactly 30 eggs were collected from cage populations (which were maintained under LD12:12) and placed into long glass vials (19 cm × 2.5 cm) and for each strain, 10 replicate vials were prepared. Though we started with 10 vials and 30 eggs in each of them, during analysis, vials with very low survivorship (< 33%) were removed. Therefore, ‘n’ as mentioned in Figure Legends represents the number of vials used for analysis. Five separate experimental regimens were used namely: T20 (LD10:10), T24 (LD12:12), T28 (LD 14:14), DD and LL. For all regimes eggs were collected at ZT4 under LD12:12 where ZT0 is the time of lights-on for all experiments.

Pupation time was estimated only under T20, T24 and T28 regimes, where the vials were inspected once every two hours after the first indication of pupation and the number of pupae were counted. Time to pupation was calculated as the duration between the mid-point of egg collection and the formation of puparium for each individual fly. Further, for all the five regimes examined, once the pigmentation stage for all pupae was complete, vials were inspected every two hours and the freshly emerged adults (over the past 2 h) were separated and counted giving the total number of emerged flies (without distinguishing between sexes). Time to emergence was calculated as the duration between the mid-point of egg collection and the emergence of an adult fly from the pupa.

### Pupation rhythmicity assay

Approximately 300 eggs were collected from cage populations of a wild type strain which is an ancestor of the control *per*^*+*^ flies, CP1, [28] into long glass vials (19 cm × 2.5 cm) and for each strain, 10 replicate vials were placed under T24 (LD 12:12). Once larvae began to pupate, vials were observed manually every two hours to count the number of pupae. A dim far red (> 650 nm) lamp was used during the dark phase. Pupation profiles were plotted by averaging the number of larvae pupated across vials across days for each time point.

### Adult-emergence rhythm assay to determine periodicity of emergence

Approximately 300 eggs were collected from cage populations into long glass vials (19 cm × 2.5 cm) and for each strain, 10 replicate vials were placed in each of the T-cycles i.e., T24 (LD12:12), T20 (LD10:10) and T28 (LD14:14). Although we started with 10 vials and ~ 300 eggs in each of them, during analysis, vials in which the number of flies dropped to less than 20 per cycle were not considered. Therefore ‘n’ as mentioned in Figure Legends represents the number of vials used for analysis. Once the pigmentation stage for all pupae was complete, observations were made every two hours to count the number of emerged flies (males+females). Adult emergence profiles were plotted by averaging the number of flies emerged across vials across days for each time point. We were able to obtain a maximum of 3–4 cycles for our flies and therefore it was not possible to have free-running rhythm data.

### Statistical test

Since the development profiles for all strains were skewed, we chose median rather than mean to assess the differences. Median time to emergence was estimated for each vial for each strain by marking the time at which 50% or more emergence had occurred. Similarly, median pupation time was estimated for each vial for each strain by marking the time at which 50% or more pupation had occurred. Kruskal-Wallis test was performed for comparison of medians obtained from the three strains in each regime examined as all the data sets did not conform to normality as determined by Shapiro-Wilk test. For pupation rhythm and adult emergence rhythm assays, percentage pupation and emergence respectively were calculated at every time point for each cycle and one-way ANOVA was performed over mean values across vials to examine the effect of time point. Further, we estimated periodicity over 3–4 cycles for each vial using COSINOR analysis in Microsoft Excel (version 2010). The time series obtained for a given vial was fitted with a cosine curve [M- (Acoswt + ψ), M being the mesor for time series for the vial, ψ being the phase and A being the amplitude] with a specific period, phase and amplitude. That combination of period and amplitude giving minimum mismatch value (least sum of square deviations) was considered as the periodicity of that vial. If this estimated period was significantly different from period of entraining regime using the one-sample t-test at *p* < 0.05, flies of that strain were considered as not entrained. For example, to test whether eclosion rhythm of *per*^*+*^ flies in vials 1–10 were entrained to T20, one-sample t-test was done between the period values for the 10 vials (obtained as described above) against the value of 20. For all analyses, statistically significant differences were determined at *p* < 0.05. It must be noted that if flies of a given vial did not entrain to a regime, it may not necessarily mean that they were free-running as they could sometimes adopt a period far from their own intrinsic period, and that it could also include phenomena such as relative co-ordination.

## Additional files

Additional file 1: Scheme employed for backcross. Females from the mutant line *per*^*s*^ (top) and *per*^*l*^ (bottom) were crossed with males sampled from the wild-type population. Individuals from F1 generation were allowed to interbreed. Progeny from this cross were screened for homozygous mutant females by assessing free-running period of individual flies using DAM monitor system. These females (n ranging from 10 to 100) were then utilized for the next generation of backcrossing with wild-type males. Virgin females were collected for setting up each cross. Flies were allowed to mate for 2–3 days and then adults were discarded. This process was continued for 10 generations and experiments were conducted from flies sampled at 5th, 7th and 10th generation. (PDF 416 kb)

Additional file 2: Frequency distribution of free-running period. Histogram of free-running period estimated for individual flies after 7 generations of backcrossing using chi-square periodogram. The inverted brackets indicate the range of flies designated as each of the three genotypes. Mean free-running period for each genotype is shown in parentheses. (PDF 138 kb)

Additional file 3: Emergence time for male and female flies. Box plots of median time to emergence for each strain, for males **(a)** and females **(b)** (*n* > 8 vials) for the three strains assayed under T24. All other details are similar to Fig. 1. (PDF 206 kb)

Additional file 4: Emergence time for *per*^*0*^ flies. Box plots for median time to emergence for *per*^*0*^ and *per*^***+***^ flies (*n* = 10 vials; 300 eggs/vial) when assayed under T24. All other details are similar to Fig. 1. Asterisk shows that *per*^*0*^ differs significantly from *per*^*+*^ (*p* < 0.05) based on Kruskal-Wallis test for multiple independent samples. (PDF 119 kb)

Additional file 5: Adult emergence time series under T24. Adult-emergence profiles of the three strains (n = 10 vials; 300 eggs/vial) (top: *per*^*s*^; middle: *per*^*+*^; bottom: *per*^*l*^) under T24 across consecutive cycles where percentage emergence is plotted against Zeitgeber time, 0 being the time of lights-on for each cycle. Shaded regions represent duration of the LD cycle during which lights were off. Error bars are SEM measured across replicate vials (n = 10). (PDF 376 kb)

Additional file 6: Adult emergence time series under T20. Adult-emergence profiles of the three strains (n = 10; 300 eggs/vial) (top: *per*^*s*^; middle: *per*^*+*^; bottom: *per*^*l*^) under T20 across consecutive cycles where percentage emergence is plotted against Zeitgeber time, 0 being the time of lights-on for each cycle. Shaded regions represent duration of the LD cycle during which lights were off. Error bars are SEM measured across replicate vials (n = 10). (PDF 396 kb)

Additional file 7: Adult-emergence time series under T28. Adult-emergence profiles of the three strains (*n* = 8; 300 eggs/vial) (top: *per*^*s*^; middle: *per*^*+*^; bottom: *per*^*l*^) under T28 across consecutive cycles where percentage emergence is plotted against Zeitgeber time, 0 being the time of lights-on for each cycle. Shaded regions represent duration of the LD cycle during which lights were off. Error bars are SEM measured across replicate vials (n = 8). (PDF 453 kb)

Additional file 8: Pupation profile under T24. Pupation profiles of wild type flies (n = 8; 300 eggs/vial) under T24 where percentage pupation is plotted against Zeitgeber time across cycles, 0 being the time of lights-on for each cycle. Shaded regions represent duration of the LD cycle during which lights were off. Error bars are SEM measured across replicate vials (n = 8). (PDF 179 kb)

## Abbreviations

DD: Constant darkness
LD: Light-dark cycle
LL: Constant light
T20: Light-dark cycle with 10 hours of light and 10 hours of dark.
T24: Light-dark cycle with 12 hours of light and 12 hours of dark.
T28: Light-dark cycle with 14 hours of light and 14 hours of dark
