# Supplementary figures and images for: Environmental cycles regulate development time via circadian clock mediated gating of adult emergence
Source: BMC Dev Biol. 2018 Dec 22;18:21. doi: 10.1186/s12861-018-0180-6 (PMC6303858; doi:10.1186/s12861-018-0180-6)

a

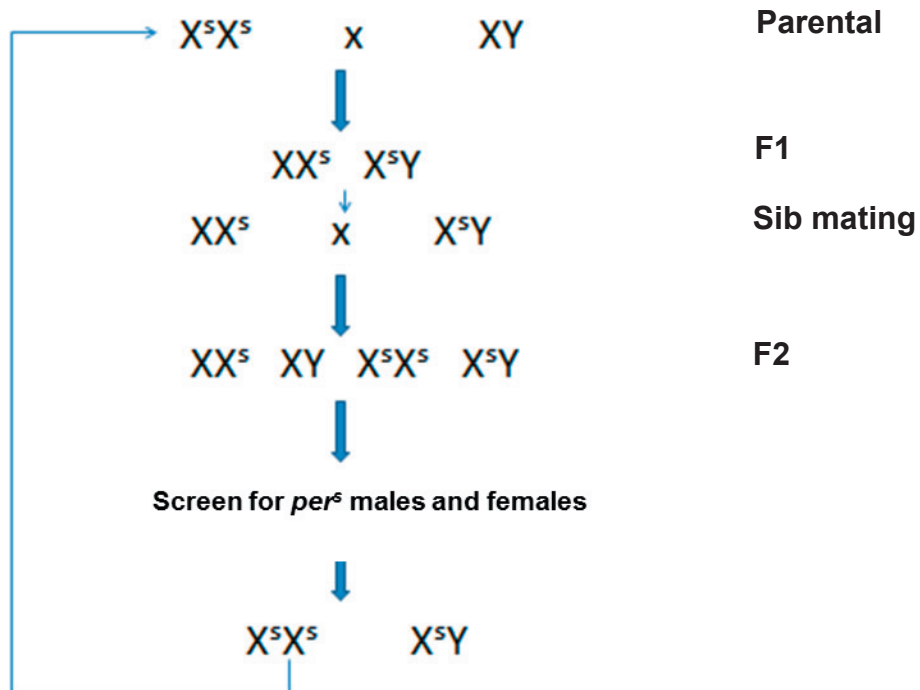

b

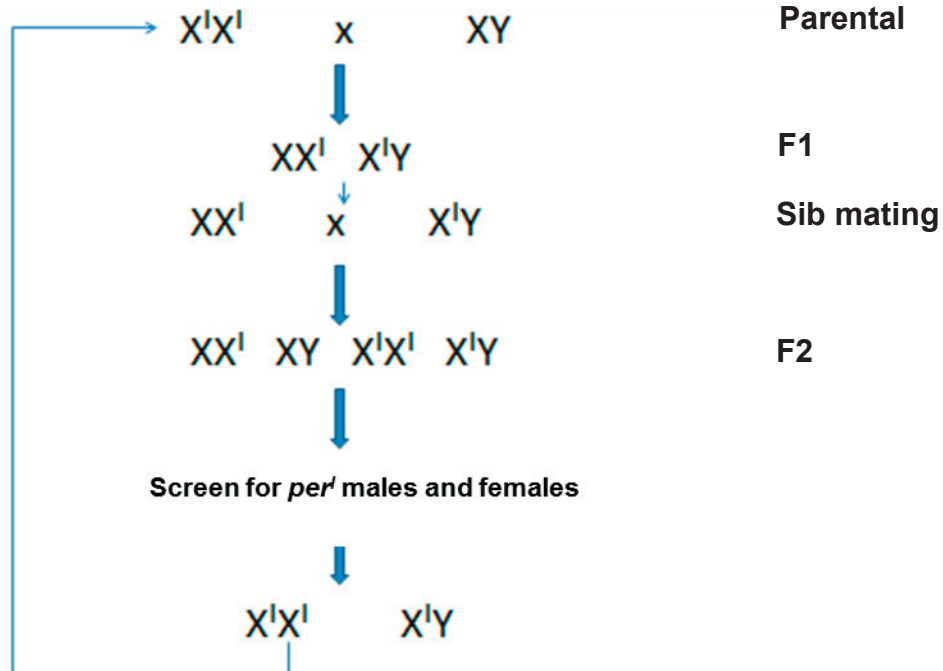

Supplement: Supplementary file 1 — Scheme employed for backcross. Females from the mutant line pers (top) and perl (bottom) were crossed with males sampled from the wild-type population. Individuals from F1 generation were allowed to interbreed. Progeny from this cross were screened for homozygous mutant females by assessing free-running period of individual flies using DAM monitor system. These females (n ranging from 10 to 100) were then utilized for the next generation of backcrossing with wild-type males. Virgin females were collected for setting up each cross. Flies were allowed to mate for 2–3 days and then adults were discarded. This process was continued for 10 generations and experiments were conducted from flies sampled at 5th, 7th and 10th generation. (PDF 416 kb) [file 12861_2018_180_MOESM1_ESM.pdf]

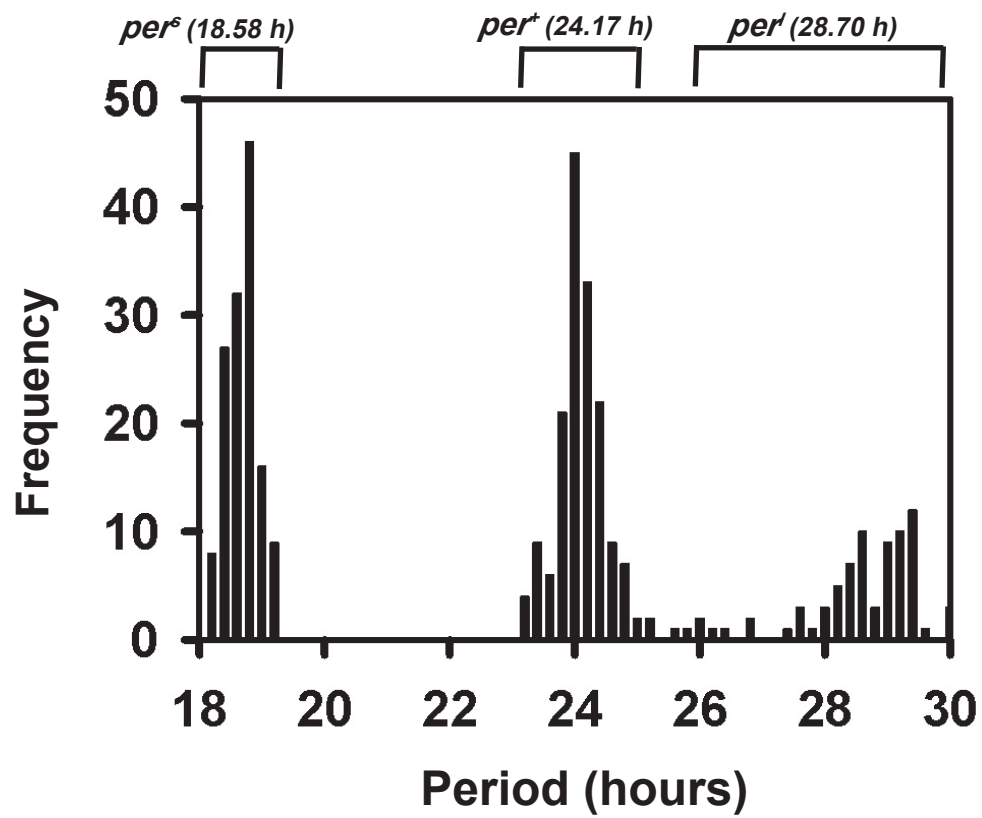

Supplement: Supplementary file 2 — Frequency distribution of free-running period. Histogram of free-running period estimated for individual flies after 7 generations of backcrossing using chi-square periodogram. The inverted brackets indicate the range of flies designated as each of the three genotypes. Mean free-running period for each genotype is shown in parentheses. (PDF 138 kb) [file 12861_2018_180_MOESM2_ESM.pdf]

**a**

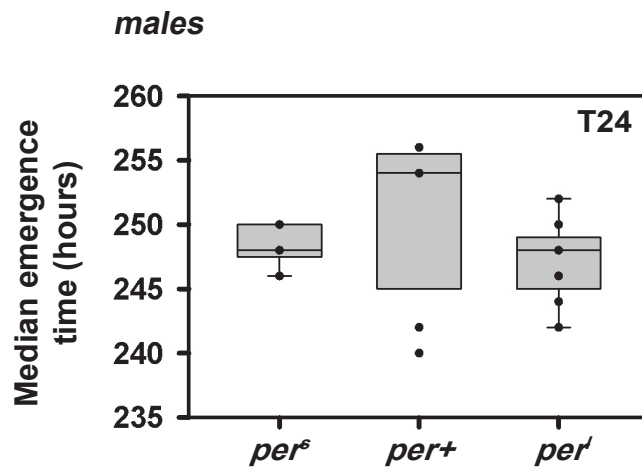

**b**

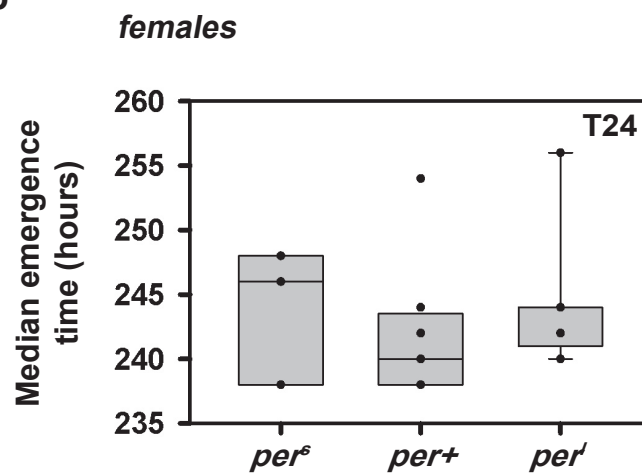

Supplement: Supplementary file 3 — Emergence time for male and female flies. Box plots of median time to emergence for each strain, for males (a) and females (b) (n > 8 vials) for the three strains assayed under T24. All other details are similar to Fig. 1. (PDF 206 kb) [file 12861_2018_180_MOESM3_ESM.pdf]

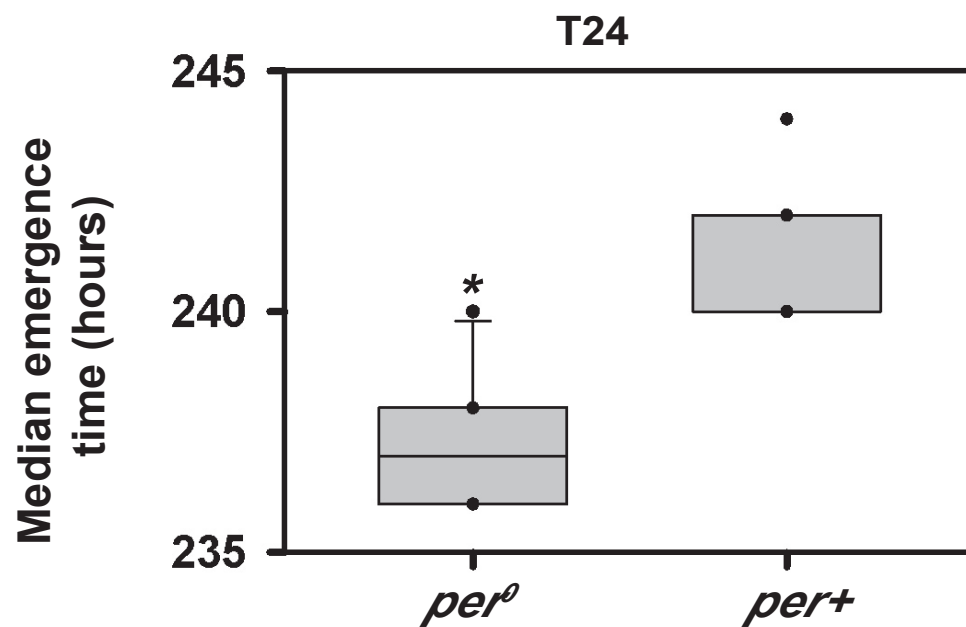

Supplement: Supplementary file 4 — Emergence time for per0 flies. Box plots for median time to emergence for per0 and per+ flies (n = 10 vials; 300 eggs/vial) when assayed under T24. All other details are similar to Fig. 1. Asterisk shows that per0 differs significantly from per+ (p < 0.05) based on Kruskal-Wallis test for multiple independent samples. (PDF 119 kb) [file 12861_2018_180_MOESM4_ESM.pdf]

T24

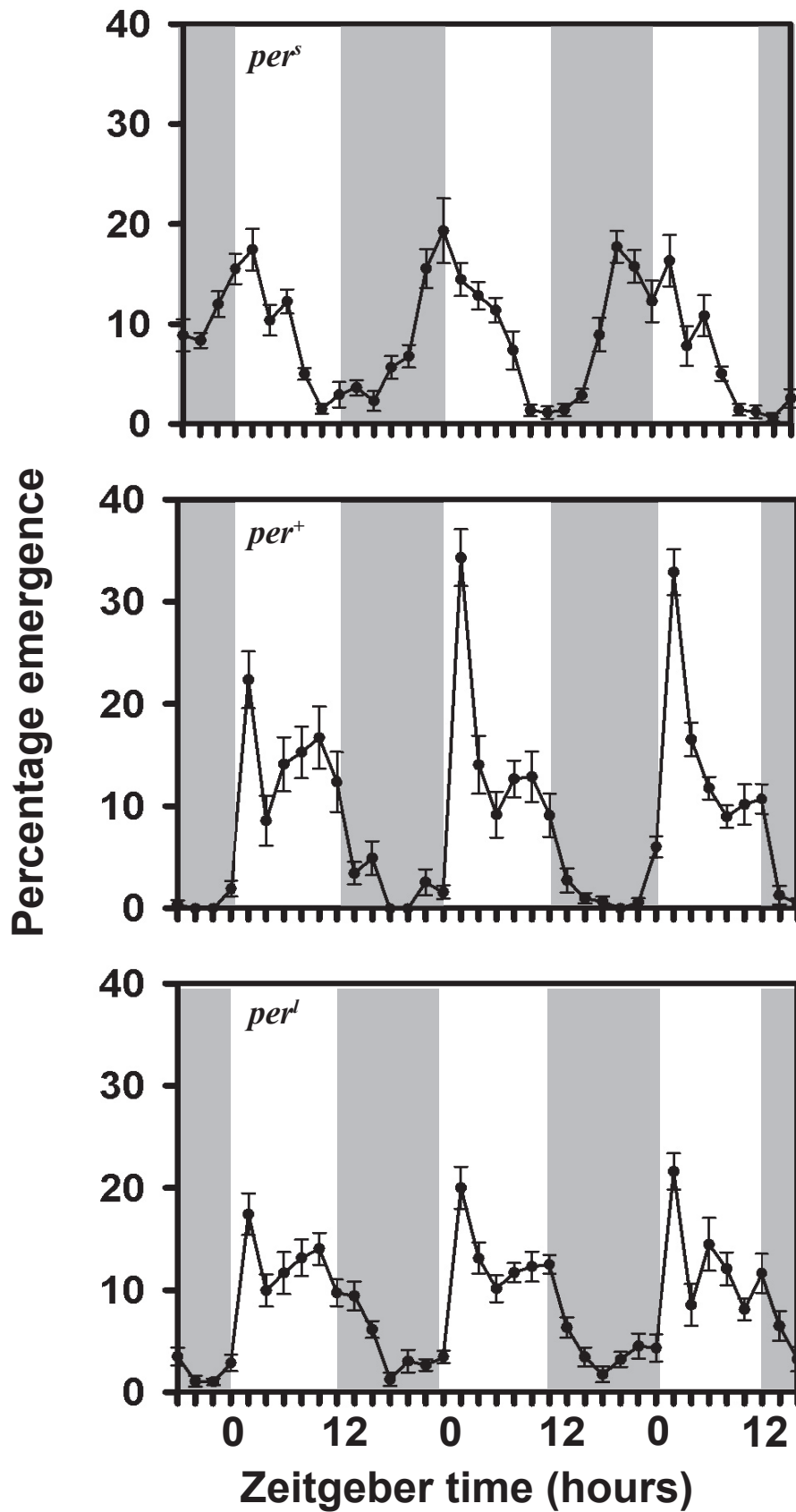

Supplement: Supplementary file 5 — Adult emergence time series under T24. Adult-emergence profiles of the three strains (n = 10 vials; 300 eggs/vial) (top: pers; middle: per+; bottom: perl) under T24 across consecutive cycles where percentage emergence is plotted against Zeitgeber time, 0 being the time of lights-on for each cycle. Shaded regions represent duration of the LD cycle during which lights were off. Error bars are SEM measured across replicate vials (n = 10). (PDF 376 kb) [file 12861_2018_180_MOESM5_ESM.pdf]

T20

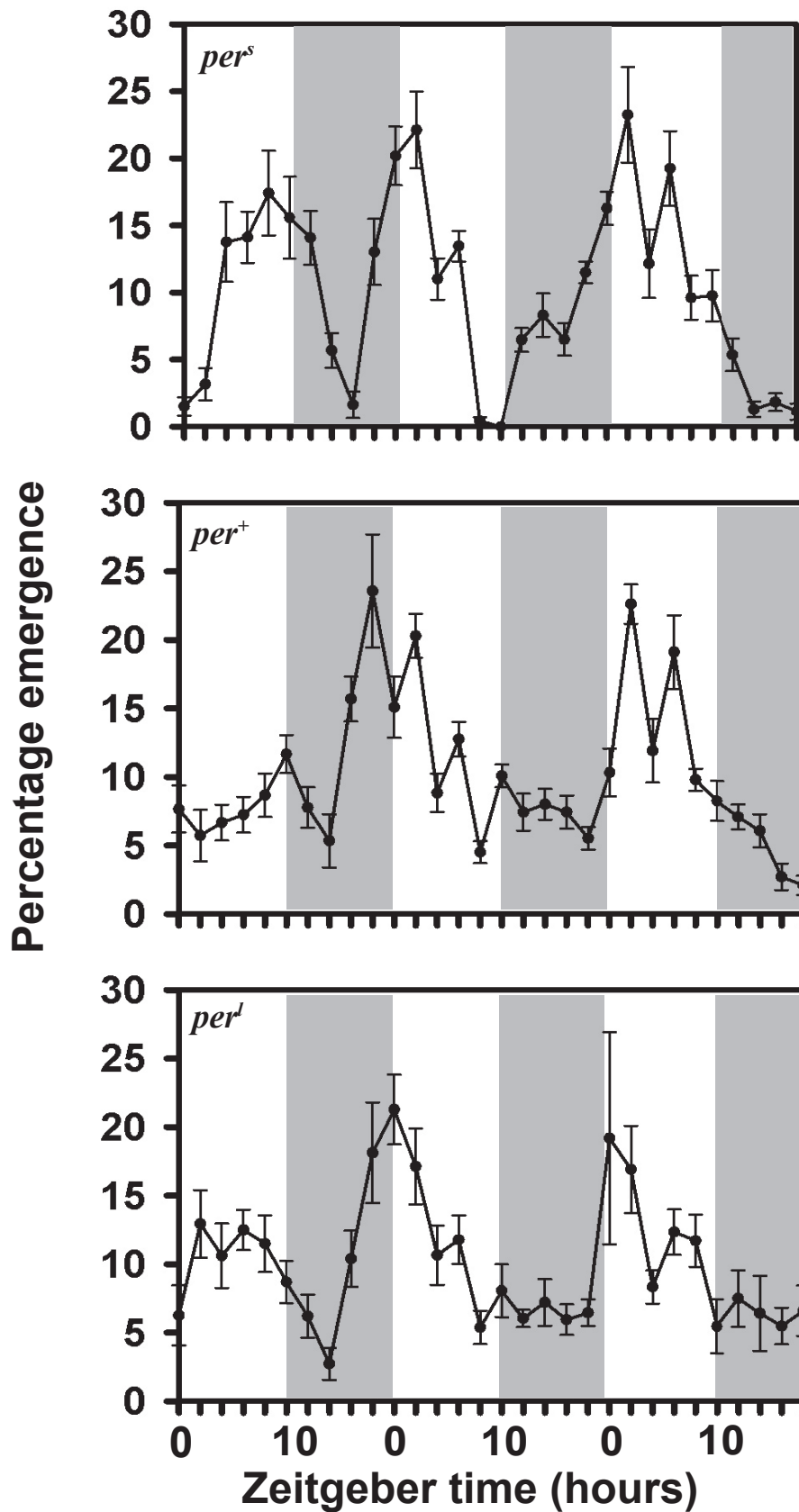

Supplement: Supplementary file 6 — Adult emergence time series under T20. Adult-emergence profiles of the three strains (n = 10; 300 eggs/vial) (top: pers; middle: per+; bottom: perl) under T20 across consecutive cycles where percentage emergence is plotted against Zeitgeber time, 0 being the time of lights-on for each cycle. Shaded regions represent duration of the LD cycle during which lights were off. Error bars are SEM measured across replicate vials (n = 10). (PDF 396 kb) [file 12861_2018_180_MOESM6_ESM.pdf]

T28

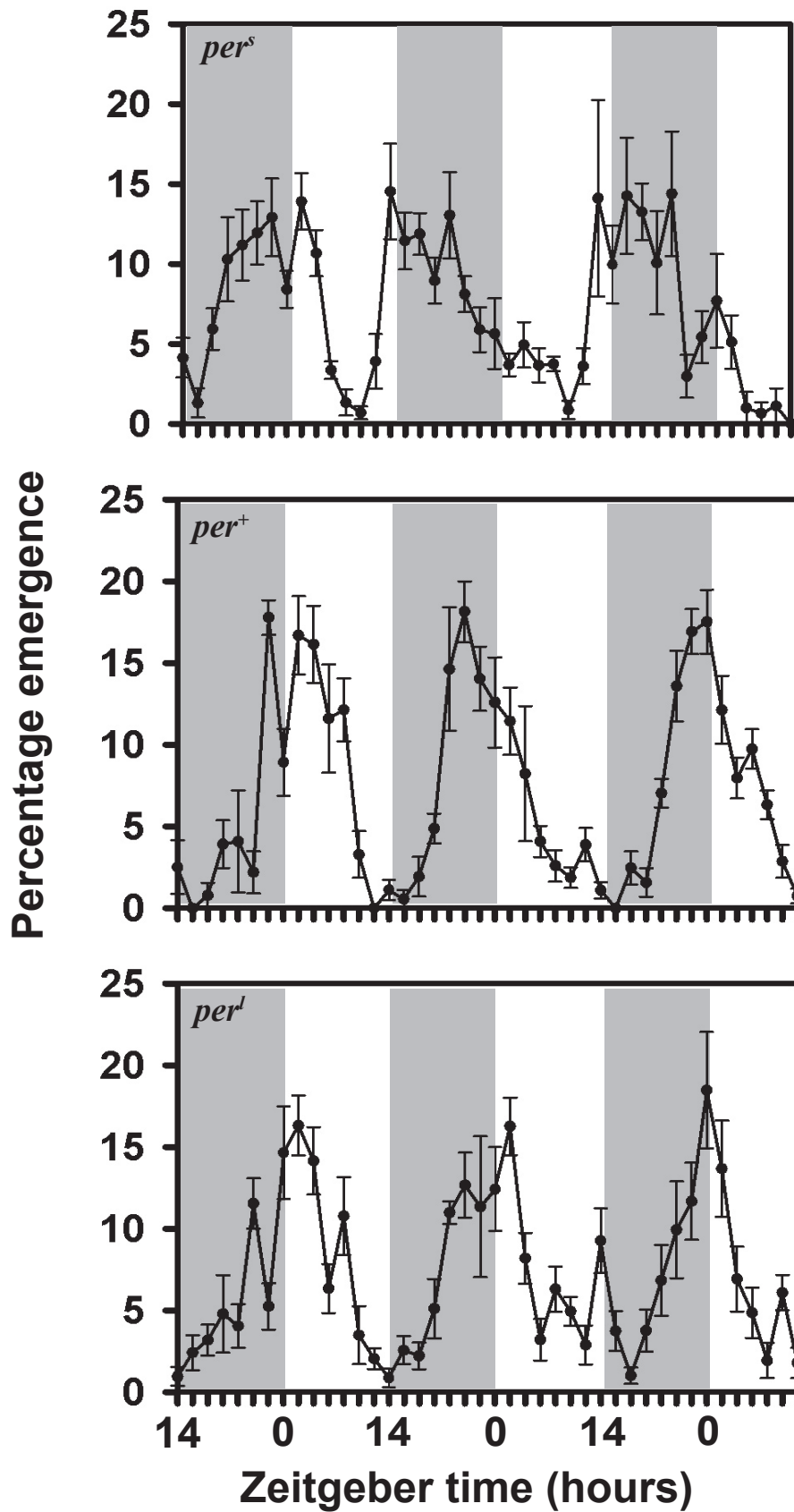

Supplement: Supplementary file 7 — Adult-emergence time series under T28. Adult-emergence profiles of the three strains (n = 8; 300 eggs/vial) (top: pers; middle: per+; bottom: perl) under T28 across consecutive cycles where percentage emergence is plotted against Zeitgeber time, 0 being the time of lights-on for each cycle. Shaded regions represent duration of the LD cycle during which lights were off. Error bars are SEM measured across replicate vials (n = 8). (PDF 453 kb) [file 12861_2018_180_MOESM7_ESM.pdf]

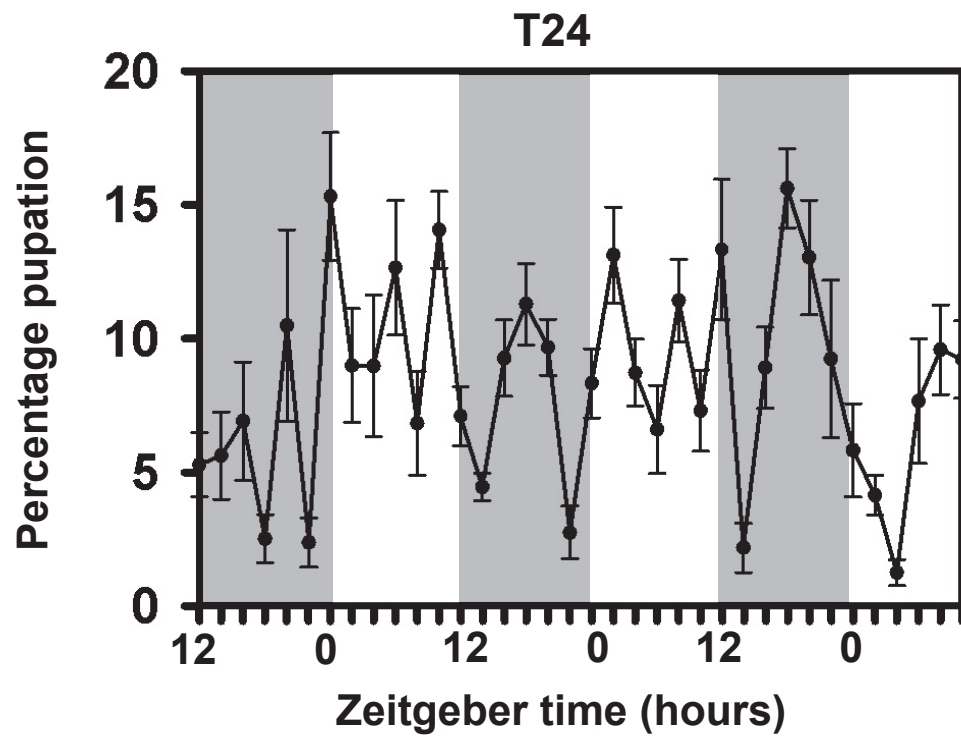

Supplement: Supplementary file 8 — Pupation profile under T24. Pupation profiles of wild type flies (n = 8; 300 eggs/vial) under T24 where percentage pupation is plotted against Zeitgeber time across cycles, 0 being the time of lights-on for each cycle. Shaded regions represent duration of the LD cycle during which lights were off. Error bars are SEM measured across replicate vials (n = 8). (PDF 179 kb) [file 12861_2018_180_MOESM8_ESM.pdf]
